# Clinically Approved Iron Chelators Influence Zebrafish Mortality, Hatching Morphology and Cardiac Function

**DOI:** 10.1371/journal.pone.0109880

**Published:** 2014-10-16

**Authors:** Jasmine L. Hamilton, Azadeh Hatef, Muhammad Imran ul-haq, Neelima Nair, Suraj Unniappan, Jayachandran N. Kizhakkedathu

**Affiliations:** 1 The Centre for Blood Research, Department of Pathology and Laboratory Medicine, Life Sciences Institute, The University of British Columbia, Vancouver, British Columbia, Canada; 2 Veterinary Biomedical Sciences, Laboratory of Integrative Neuroendocrinology, Western College of Veterinary Medicine, University of Saskatchewan, Saskatoon, Saskatchewan, Canada; 3 Department of Chemistry, The University of British Columbia, Vancouver, British Columbia, Canada; University of Sao Paulo - USP, Brazil

## Abstract

Iron chelation therapy using iron (III) specific chelators such as desferrioxamine (DFO, Desferal), deferasirox (Exjade or ICL-670), and deferiprone (Ferriprox or L1) are the current standard of care for the treatment of iron overload. Although each chelator is capable of promoting some degree of iron excretion, these chelators are also associated with a wide range of well documented toxicities. However, there is currently very limited data available on their effects in developing embryos. In this study, we took advantage of the rapid development and transparency of the zebrafish embryo, *Danio rerio* to assess and compare the toxicity of iron chelators. All three iron chelators described above were delivered to zebrafish embryos by direct soaking and their effects on mortality, hatching and developmental morphology were monitored for 96 hpf. To determine whether toxicity was specific to embryos, we examined the effects of chelator exposure via intra peritoneal injection on the cardiac function and gene expression in adult zebrafish. Chelators varied significantly in their effects on embryo mortality, hatching and morphology. While none of the embryos or adults exposed to DFO were negatively affected, ICL -treated embryos and adults differed significantly from controls, and L1 exerted toxic effects in embryos alone. ICL-670 significantly increased the mortality of embryos treated with doses of 0.25 mM or higher and also affected embryo morphology, causing curvature of larvae treated with concentrations above 0.5 mM. ICL-670 exposure (10 µL of 0.1 mM injection) also significantly increased the heart rate and cardiac output of adult zebrafish. While L1 exposure did not cause toxicity in adults, it did cause morphological defects in embryos at 0.5 mM. This study provides first evidence on iron chelator toxicity in early development and will help to guide our approach on better understanding the mechanism of iron chelator toxicity.

## Introduction

Iron chelators are used to treat transfusion associated iron overload in patients with β-thalassemia (TM), sickle-cell anemia (SCD) and myelodysplastic syndromes (MDS) [1]–[2]), and for the treatment of metal poisoning in children [3]. Due to ineffective erythropoiesis, patients with TM and to a lesser extent, SCD, must be treated with red blood cell (RBC) transfusions to ameliorate anemia. RBC transfusions also reduce the risk of stroke in patients with SCD and are used as supportive care for treating anemia in MDS [2], [4]. However, due to the high iron content in RBCs and the inability of man to actively excrete iron, transfusion therapy inevitably leads to the development of iron overload. The long term adverse consequences of iron overload are numerous; ranging from growth retardation in children to iron induced cardiac dysfunction, the life-limiting complication of severe iron overload [5]. Thus, the degree of iron loading is correlated to life expectancy.

**Figure 1:**
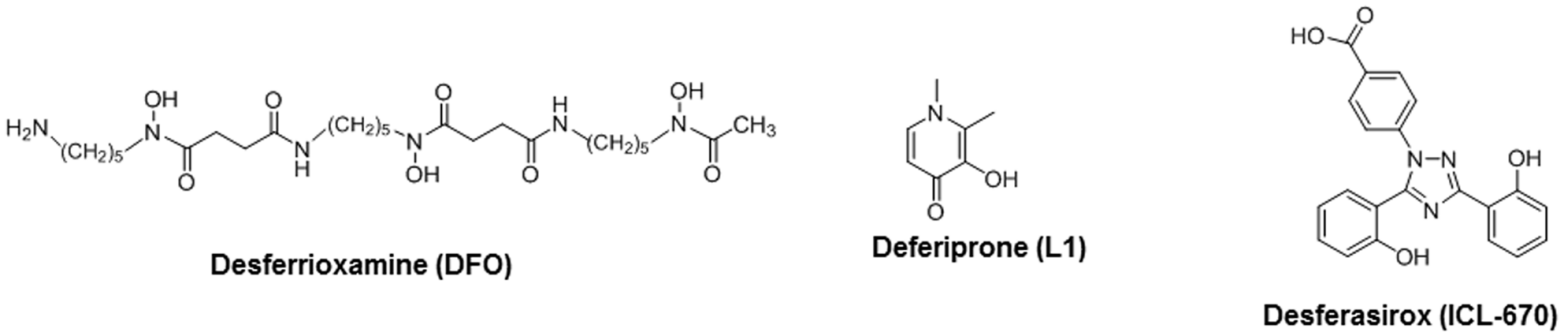
The chemical structures of clinically approved iron chelators.

Iron chelation therapy (ICT) is used to manage iron overload [6]. Iron chelators bind iron to form non-toxic complexes that are then excreted from the body, enabling safer body iron levels [6]–[7]. There are 3 chelators currently approved for treating transfusion associated iron overload (**Figure 1**). Desferrioxamine (DFO), the oldest iron chelator, has been used in clinical practice since the 1960s and has drastically improved survival in TM patients who comply with therapy [8]. Deferiprone (L1), the second iron chelator to be licensed and the first orally active chelator to become available is indicated for use when chelation with DFO alone does not work well enough [9]. Unlike DFO, which has a very low cellular permeability, L1 is reported to have an ability to enter cardiac cells and has proven to be efficacious in enhancing iron excretion when used in combination with DFO [10]. Desferasirox (ICL-670) is the second orally active iron chelator to become available and the most recent to be approved for use [11]–[12]. Like L1, studies suggest that ICL-670 can also penetrate cells to access intracellular iron pools and has positively contributed to patient adherence to therapy [12]. Further details regarding the properties of DFO, L1 and ICL-670 are given in **Table 1**.

**Table 1:** Summary of the properties of iron chelators in clinical use.

Property of Chelator	Desferrioxamine (DFO)	Deferiprone (L1)	Desferasirox (ICL-670)
**Date of approval for clinical use^1,8,14^**	1970s	1999 in Europe and Asia,2012 in the USA	2005
**Usual dose^1^**	20–50 mg/kg/day	75–100 mg/kg/day	20–40 mg/kg/day
**Molecular weight**	560	139	373
**Fe binding log stability constant^6,7,32^**	30.6	35	38
**Chelator: Iron^39^**	1∶1 (Hexadentate)	3∶1 (Bidentate)	2∶1 (Tridentate)
**Potential toxicities^11,13,16^**	Reaction at the infusion site,neurotoxicity, boneabnormalities	Neutropenia, agranulocytosis,arthralgia, elevation of liverenzyme	Gastrointestinal, rash, renal and liver

Iron chelators can reduce complications such as cardiomyopathy, the major cause of death from iron overload. Further, iron chelation therapy can slow the progression of liver fibrosis and reduce glucose intolerance in transfusion dependent patients [5]–[6]. However, a wide range of chelator-induced toxicities have also been reported. The use of DFO at high doses may cause neurological disturbances, growth retardation, peripheral neuropathies, vision changes, endocrine dysfuction and bone deformities [13]–[14]. Severe toxicities associated with L1 include agranulocytosis and neutropenia [14]–[16]. Gastrointestinal disturbances, arthropathy and increased liver-enzyme levels were also reported [15]. ICL-670 can cause severe renal impairment, and failure, increase in serum creatinine level as well as gastrointestinal hemorrhage in some patients [17]–[18].

While toxicities of iron chelators are well documented, there is currently very limited data regarding the toxicity of DFO, ICL-670 and L1 in developing embryos. A few studies have been conducted in mice and show that DFO caused developmental toxicity only in the presence of maternal toxicity and that L1 can cause toxicity in rat embryos [19]–[21]. Thus, in this study we took advantage of the optical transparency and ex-utero development of zebrafish embryos to directly assess the functional, morphological and behavioral effects of clinically approved iron chelators on embryos in the absence of interference from maternal factors.

Zebrafish (*Danio rerio*) are increasingly used as a cost-effective *in vivo* model and have been shown to be useful in evaluating chemical toxicity [22]–[24]. The toxicity data obtained in zebrafish correlates well with developmental toxicity data from rat *in vivo* studies and previous studies have demonstrated that the prediction success rate for some drugs can be as high as 100% in zebrafish [25]–[26]. Further advantages of zebrafish include the rapid rate of organogenesis and the large number of embryos obtained per spawn, which allows throughput screening [27].

In this study, we exposed developing zebrafish embryos to various concentrations of DFO, L1 and ICL-670 and assessed their effects on mortality, hatching and morphology in embryos. To determine whether the effects were specific to embryos and to probe possible underlying mechanisms of the observed toxicities, we investigated the effects of chelator exposure on the cardiac function and gene expression in adults. Since the toxicity of iron chelators can result from sequestration of iron required for biological processes, we investigated the change in expression of hepcidin, ferroportin (FPN) and DMT-1, three genes directly involved in iron transport and recycling [28]–[31]. This study improves our understanding of the differences in potential effects of clinically approved iron chelators at the earlier stages of embryonic development. Results will guide our approach in further understanding the effect of iron chelation in vertebrates during development, and will provide clues on the possible organ systems perturbed by iron depletion in developing embryos.

## Materials and Methods

### Chemicals

Desferrioxamine (>99%), deferiprone (1, 2-dimethyl, 3-hydroxy, pyrid-4-one) and 100% cell culture grade DMSO were obtained from Sigma-Aldrich Canada Ltd. (Oakville, ON). Desferasirox (4-[3, 5-bis (2-hydroxyphenyl)-1,2,4-triazol-1-yl]benzoic acid) was synthesized in house according to established protocols [32]. The analysis and characterization of the final product are shown in supplementary materials (Figures S1 and S2 in File S1). Aquacalm was obtained from Syndel Laboratories Ltd. (British Columbia, Canada).

### Zebrafish Husbandry

All experiments complied with the Canadian Council of Animal Care guidelines and the animal care protocol (2012-0082), which was approved by the University of Saskatchewan Animal Research Ethics Board. Zebrafish were obtained from (Aquatic Imports, Calgary) and were maintained in a recirculating, light and temperature controlled facility on a standard 14∶10 h light:dark cycle in standard system fish water. Embryos were maintained at 28°C throughout all experiments. Embryos were washed and healthy, non-coagulated embryos were selected for exposure to chelators.

### Chelator Exposure to Zebrafish Embryos

Embryos were grown in 6-well plates (Corning, Life sciences) containing 4 mL of chelator solution in DMSO and serial dilutions were done using fish water. DMSO was used as control. Chelator solutions were replenished every day and seven concentrations of chelator (0.015, 0.03, 0.06, 0.125, 0.25, 0.5 and 1 mM) were used. The maximum DMSO concentration for DFO and ICL-670 was 0.45%. The maximum concentration tested for L1 was 0.5 mM, with a DMSO concentration range of 0.02–1% as shown in supplementary materials (Figure S5 in File S1). All embryos were derived from the same spawn of eggs to allow statistical comparison. After fertilization, embryos were collected and transferred to 6-well plates; 15 embryos per well and 3 wells per concentration (n = 45) for a treatment period of 96 h post fertilization (hpf). This study was repeated twice after an initial optimization study and the final concentration of DMSO present in each solution was noted and is given in supplementary materials (Figure S3–S5 in File S1).

### Mortality, Hatching and Morphology Readings

Mortality, hatching and morphology were assessed and recorded every 24 h using a CCD digital camera (OLYMPUS DP70, Japan) mounted on a microscope (Olympus, BX51, Japan) every 24 h from 6 hpf to 96 hpf. Morphology, mortality and hatching rate were determined. Other parameters such as defects in swim behavior were also monitored. Percentage of hatch (% hatch success) was defined as: (the number of larvae/initial number of embryos)×100.

### Chelator Exposure and Ultrasound Analysis in Adult Zebrafish

Adult fish were anesthetized with 28 mg/L aquacalm solution. Stock solutions were prepared in DMSO and serial dilutions were prepared before each experiment using saline (0.15 M NaCl). The final concentration of DMSO present in each solution was noted and is given in supplementary materials (Figure S3 in File S1). Male and female zebrafish were used and emphasis was placed on identifying the gender of each fish and balancing the number of males and females in each group. The fish used in our experiments were approximately 1 year old. Six adult zebrafish were injected intraperitoneally (*i.p.*) with 10 µL of 100 µM of each chelator and subjected to ultrasound monitoring using a VEVO 660 high frequency ultrasound machine (VisualSonics, Markham, ON), 30 minutes post injection. Each zebrafish was removed from fish water and placed in 28 mg/L aquacalm until they succumbed to the anesthetic. Once sufficiently anesthetized, they were positioned ventral side up and secured in a 3% agarose gel with minutien pins (Fine Science Tools, Vancouver, BC). The probe (A RMV 708B), attached to the machine is kept at startup mode so that the probe is already moving. The probe is then lowered above the horizontally placed zebrafish and adjusted as the view is needed. The ultrasound monitoring was carried out using the VisualSonics software (Markham, ON). Three short axis views; A1 (towards the gills), A2 (slightly away from the gills) and A3 (towards the tail), were taken. One long axis view of the ventricle was also taken by placing the fish in a vertical position. All these readings were measured both at the systole and diastole using the VisualSonics Software. Calculations were conducted as follow: Stroke Volume (SV) = EDV (end diastolic volume)−ESV (end systolic volume). Heart Rate = heart rate/10 sec×6 and cardiac output = stroke volume×heart rate.

### Quantitative PCR

Six adult zebrafish from each group were injected *i.p.* with 10 µL of 100 µM of chelators and sacrificed 24 h after injection. Liver, heart and gut were harvested for RNA extraction. Total RNA was extracted using the TRIzol RNA isolation reagent (Invitrogen, Canada).

The purity of extracted RNA was assessed by optical density absorption ratio (OD 260 nm/OD 280 nm) using the nanodrop (ND 100, NanoDrop Technologies Inc. Wilmington, DE, USA). One microgram of RNA was used for iScript cDNA synthesis as directed by the manufacturer (BioRad, Canada). cDNAs were diluted 1∶3 before qRT-PCR using a CFX connect (BioRad Laboratories Inc. Canada) with iQSYBR Green supermix (BioRad, Canada). The cDNAs were amplified using the forward and reverse primers shown in Table 2 (Sigma-Aldrich Canada Ltd). For each sample, qRT-PCR was run in duplicate to ensure consistency. The thermal profile for all reactions was 3 min at 95°C and 40 cycles of 10 s at 95°C, and 30 s at 60°C. The specificity of the amplified product in the quantitative PCR assay was determined by analyzing the melting curve to discriminate target amplicon from primer dimer and other nonspecific products. A single melt curve was observed for each primer set in all quantitative PCR reactions. Fluorescence monitoring occurred at the end of each cycle. Relative expression levels were determined by normalizing to the β-actin housekeeping gene. Results were determined using method of Livak and Schmittgen [33]. The genes investigated were hepcidin, ferroportin (FPN) and the divalent metal transporter (DMT-1) [28]–[31], [34]. Primers and accession numbers can be found in **Table 2**.

**Table 2:** The forward and reverse primers used for real-time qPCR.

Gene	Primer	Accession No. Gen Bank	Amplicon size, bp
**Hamp 1**	F: 5′-CCGAGCAGAAGACAAGTAGAT-3′R: 5′-GCAGCCAGAAACACGTTAGA-3′	NM_205583.1	**104**
**DMT-1**	F: 5′-ACCGCAGCAATAAGAAGGAG-3′R: 5′-TTGGTTTTCCCGTAGAAGGC-3′	NM_001040370.1	**136**
**Ferroportin**	F: 5′-ATTTACTTTGCCCGAGCCTT-3′R: 5′-CAGCGAGGTTTCTTTGATGC-3′	NM_131629.1	**104**
**β-Actin**	F: 5′-TTCAAACGAACGACCAACCT-3′R: 5′-TTCCGCATCCTGAGTCAATG-3′	NM_131031.1	**93**

### Statistical Analysis

Statistical analysis was performed using SPSS 19.0 software. Data were expressed as means ± SEM. Comparisons between groups were made using one-way analysis of variance (ANOVA) followed by Turkey post hoc analysis for multiple comparisons. Homogeneity of variance was tested for all data using Levene’s test. Data for non-homogeneous were log transformed to meet assumptions of normality and homoscedasticity. P values less than 0.05 were considered statistically significant.

## Results

### Effects of Clinically Used Iron Chelators on Mortality, Hatching Success and Morphology of Zebrafish Embryos

**Figure 2:**
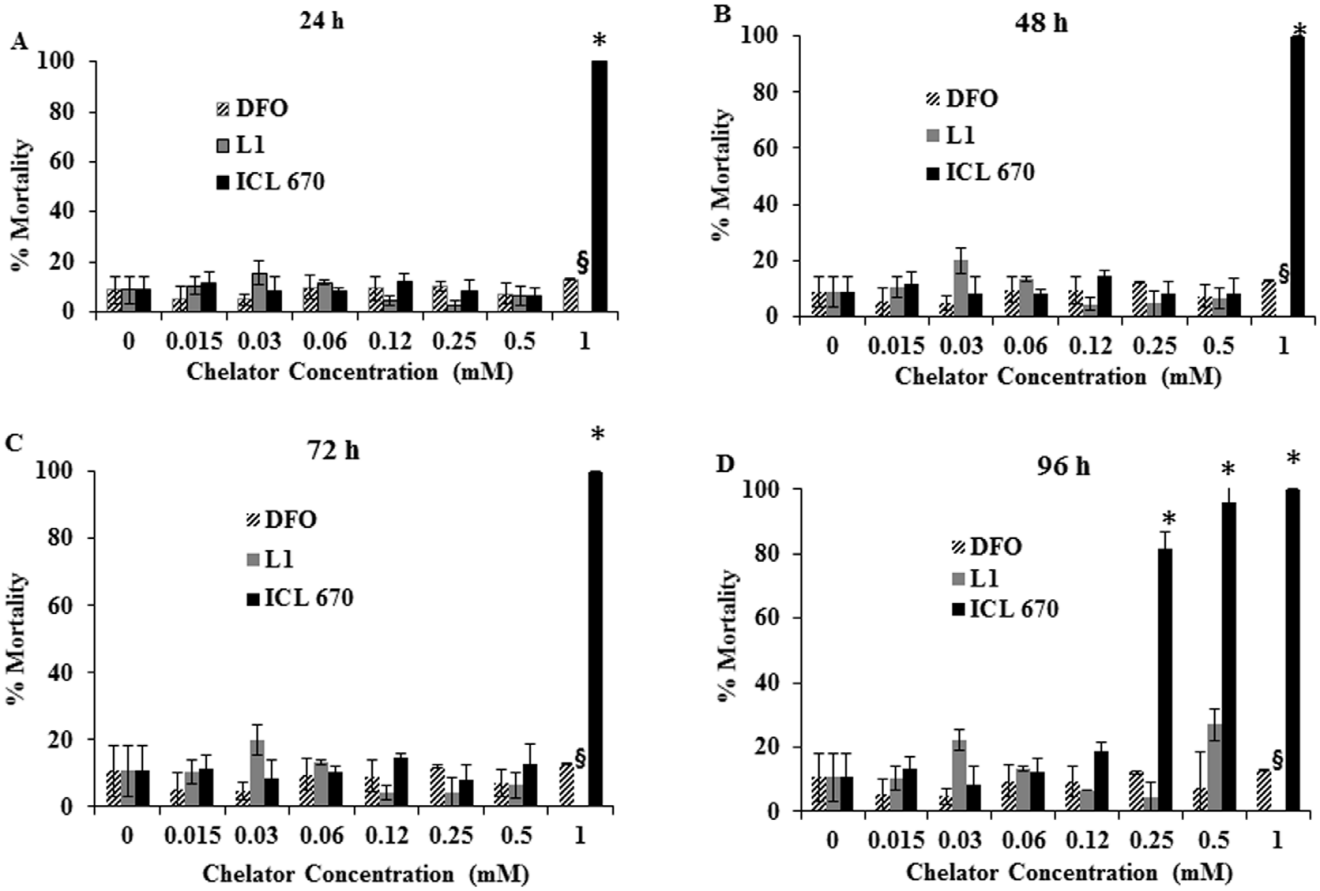
Mortality of zebrafish embryos exposed to iron chelators. The mortality of DFO and L1-treated embryos did not vary significantly from control after 96 hpf of exposure (A–D). The mortality of ICL-670-treated embryos increased in a dose and time-dependent manner and was significantly higher than control embryos; death occurred within 24 hpf of exposure to 1 mM ICL-670 (A) and 96 hpf for 0.25 and 0.5 mM treated embryos (D). **§** denotes missing bar for L1 at 1 mM; the DMSO content would have exceeded 1.5% thus, this concentration was omitted. The concentration of chelator for which mortality was significantly different from control is denoted by *.

Iron chelators (DFO, L1, ICL-670) differed significantly in their effects in developing zebrafish embryos. The mortality of zebrafish embryos exposed to different iron chelators at different time points are shown in **Figure 2**. The mortality of DFO-treated embryos did not differ significantly (p = 0.961) from the control regardless of exposure duration and drug concentration (**Figure 2**). In contrast, the mortality of ICL-670 varied significantly from controls (p<<0.05) in a dose and time-dependent manner (**Figure 2**); while the mortality of embryos exposed to L1 caused a slight but insignificant increase in mortality at 0.5 mM. The percentage mortality was highest in ICL-670 treated embryos; with more than 80% mortality occurring above 0.25 mM of ICL-670. The onset of mortality in embryos exposed to ICL-670 varied from 24 hpf for 1 mM treated embryos to 72 hpf and 96 hpf in embryos treated with 0.5 mM and 0.25 mM, respectively. L1-treated embryos did not differ significantly from control although there was a slight increase in mortality at 0.5 mM after 96 hpf exposure. Control experiments with different concentrations DMSO used for dissolving the different iron chelators suggest that there is no interference from DMSO in this study (Figure S3 in File S1). The morphology of zebrafish embryos after 48 hpf in presence of different concentrations of chelators in given in Figure S6 and Figure S7 (in File S1).

**Figure 3:**
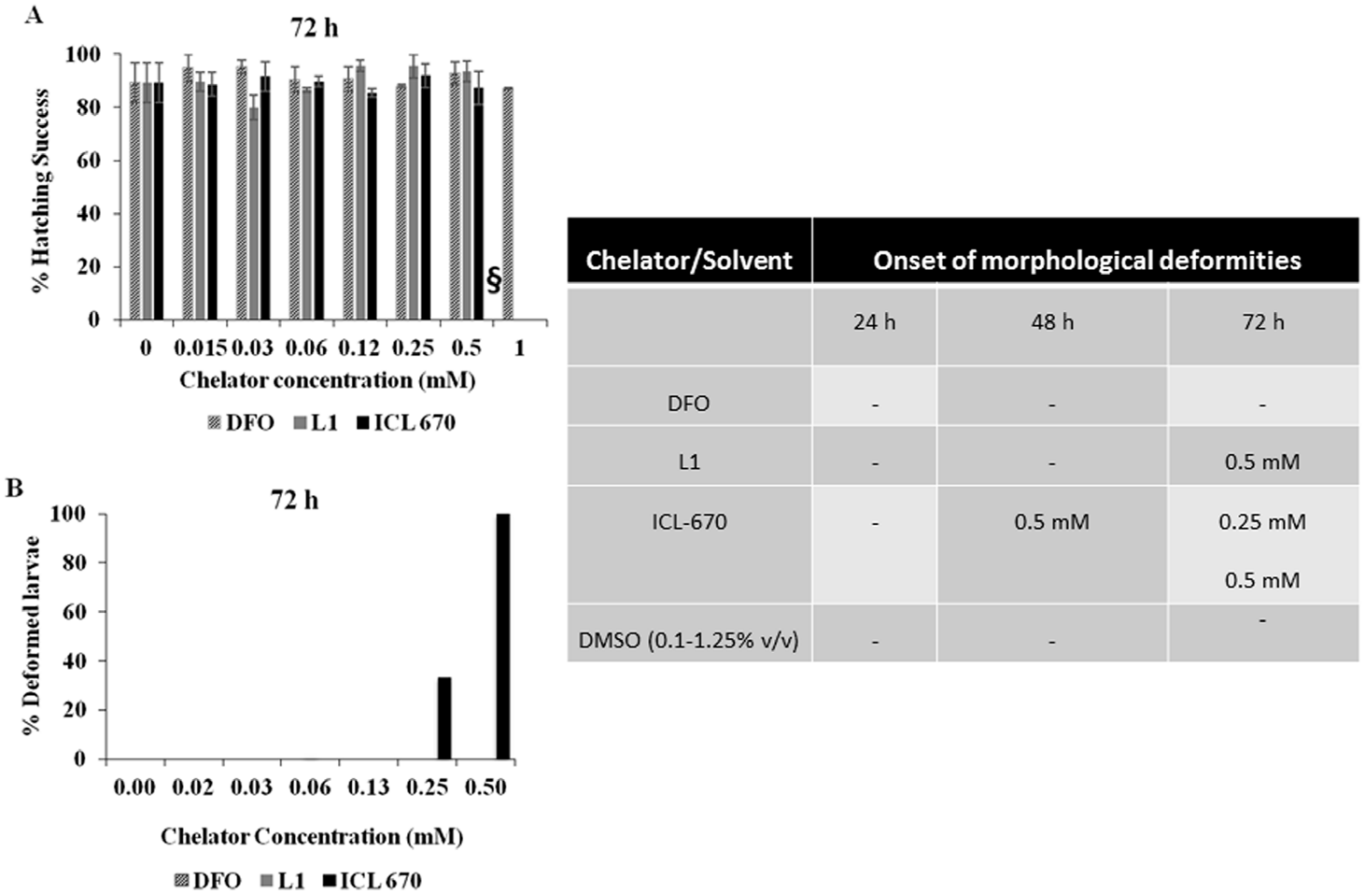
Hatching rate and morphological alterations of zebrafish embryos. DFO and L1-treated embryos hatched successfully, while ICL-670-treated embryos hatched less successfully than control due to high mortality (A). Bars missing at 1 mM are due to the death of embryos prior to hatching. **§** denoted the concentration of L1 that was excluded due to high DMSO content. There were no significant defects observed in DFO treated embryos. However, the percentage of embryos demonstrating behavioral and morphological defects increased in a time and concentration dependent manner in L1 and ICL-670-treated embryos (B). The time of onset of morphological and behavioral alterations is outlined in the figure as a separate panel. Swimming behavior was affected and bent bodies (**Figure 5**) were observed at concentrations above 0.25 mM for ICL 670-treated embryos and at 0.5 mM for L1 treated embryos.

DFO, L1 and ICL-670 also differed significantly in their effect on zebrafish embryo morphology upon hatching. Similar to the mortality, DFO-treated embryos did not show any differences in hatching when compared to control, up to concentrations of 1 mM (**Figure 3**). While all the embryos treated with L1 hatched into larvae successfully, the hatching rate was reduced in ICL-670 treated embryos due to increased mortality; embryos treated with 1 mM of ICL-670 did not survive to hatch.

**Figure 4:**
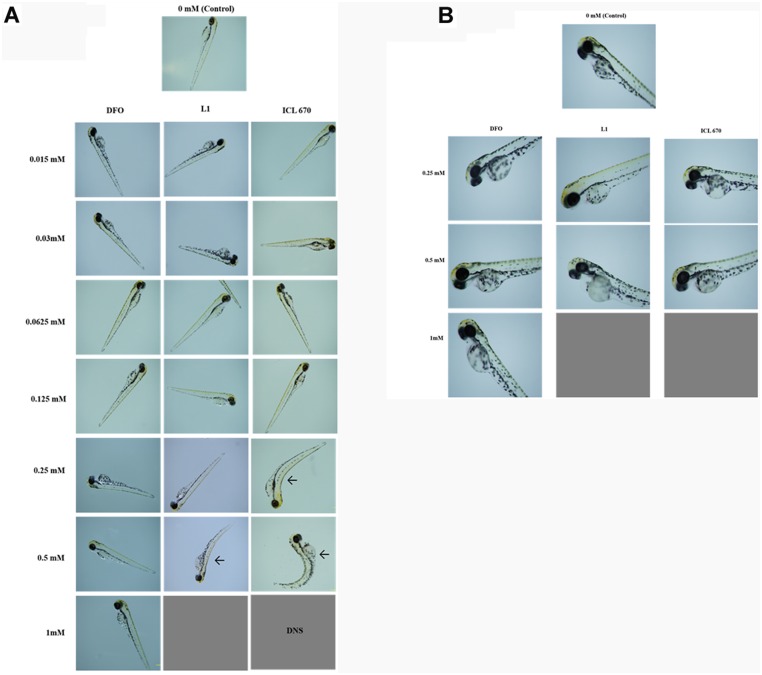
Zebrafish larvae morphology after 72 hpf of exposure to iron chelators. **A:** Representative optical images of zebrafish morphology after 72 hpf exposure to DFO, L1 and ICL 670. Zebrafish kept in DMSO was used as control. Embryos treated with 1 mM ICL-670 did not survive to hatch (DNS); while those treated with 0.25 mM and 0.5 mM hatched abnormally and had bent bodies (shown with arrows). Images were captured at 2X magnification. **B:** Representative optical images of zebrafish morphology after 72 hpf exposure to DFO, L1 and ICL 670. Higher magnification (4X) shows edema in L1 and ICL-670 treated embryos. No abnormalities were observed in DFO treated embryos up to concentrations of 1 mM. Images were captured at 4X magnification.

We next looked at the morphological deformities of the hatched larvae at different concentrations after exposure to iron chelators (**Figure 3B**). The percentage of deformities was highest for ICL-670. The deformities increased with increasing exposure time (**Figure 3C**). Representative optical images of zebrafish embryos after exposure to iron chelators and control are shown in **Figure 4**. Bent bodies (BB) of larvae were the most commonly observed defects for ICL-670 and L1. However, DFO at the doses tested, did not cause any deformities compared to controls. ICL-670 samples showed deformities at much lower concentrations than L1. Signs of rapid breathing and change in the swimming behavior were also observed. The ICL-670-treated larvae exhibited lethargy and often moved only when a small stimulus was provided. Edema was also observed in ICL-670 treated larvae. Similar but less abundant morphological defects occurred in embryos exposed to L1 at 0.5 mM (**Figures 4A and B**). Drug dose and duration of exposure were the two most important factors influencing the toxicity.

### Acute Exposure to Iron Chelators on Cardiac Output and Heart Rate in Adult Zebrafish

**Figure 5:**
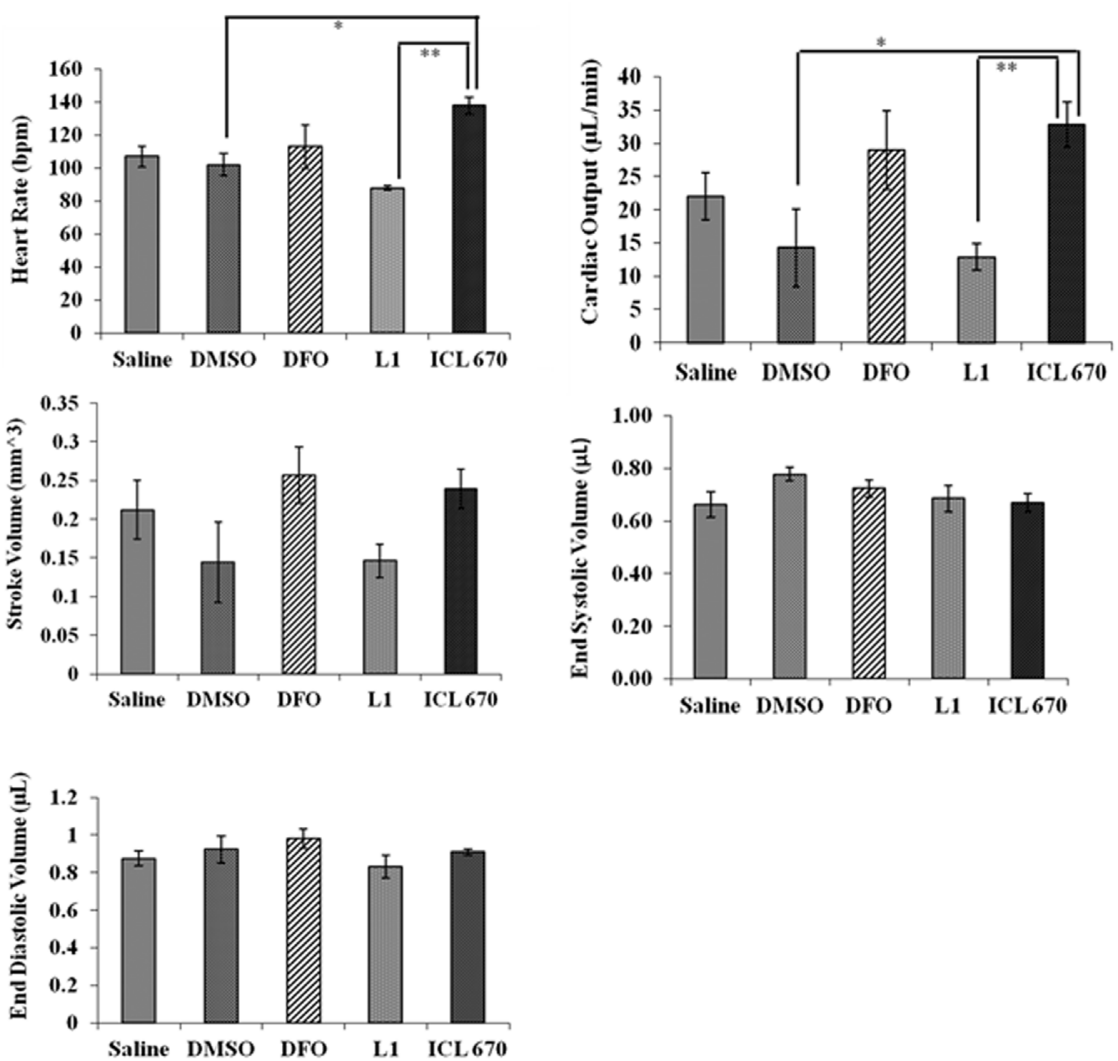
Iron chelator exposure significantly influences heart rate and cardiac output in adult zebrafish. Six adult zebrafish were injected with 10 µL of 100 µM of each chelator and subjected to analysis by ultrasound 30 min after injection. The heart rate of fish treated with ICL-670 differed significantly from controls. There was also a significant difference in heart rate between LI and ICL-670 treated fish. Similarly, the cardiac output of fish exposed to ICL-670 differed significantly from control and L1-treated fish. However, DFO and L1 treated fish did not vary significantly from controls in any of the measurements obtained. DFO, L1 and ICL-670 did not significantly affect the end-diastolic volume, end-systolic volume or the stroke volume compared to the saline or 0.1% DMSO controls.

To enhance our understanding of iron chelator-induced toxicities in zebrafish, we investigated the effects of DFO, ICL-670 and L1 on the cardiac function of adult zebrafish. Adult fish were injected with 10 µL of 100 µM of chelators and subjected to ultrasound 30 minutes after treatment. We chose a lower concentration of the chelators that was not lethal and would allow us to determine more subtle changes. The heart rate, cardiac output, stroke volume, end systolic volume and end diastolic volume were measured and are shown in **Figure 5**. We used DMSO as a vehicle control and saline treated zebrafish to confirm that the vehicle solvent did not have an adverse effect on the zebrafish and that observed effects from chelator treated fish were real. DMSO and saline treated zebrafish controls did not show any difference in heart function with aquacalm treatment. Therefore, we believe there was no unwanted effect from the anesthetic.

ICL-670 but not L1 nor DFO caused a significant increase in the heart rate and cardiac output of zebrafish (**Figure 5A and B**). The average heart rate increased from 102 to 138 beats per minute and cardiac output increased from 12 µL/min in controls compared to 30 µL/min in ICL-670, (p<0.05; P_heart rate_ = 0.001; P_cardiac output_ = 0.01). Interestingly, there was a significant difference in heart rate between L1 treated and ICL-670 treated fish. The heart rate of DFO treated fish did not differ significantly from control. Chelators did not significantly affect the end-diastolic volume, end-systolic volume and stroke volume in adult zebrafish (**Figure 5C–E**) (P_end diastolic volume_ = 0.3421; P_end systolic volume_ = 0.25; P_stroke volume_ = 0.1137).

### Acute Exposure to Iron Chelators on the Expression of Hepcidin, Ferroportin and DMT-1 Genes in Adult Zebrafish

Hepcidin, FPN and DMT-1 are genes involved in iron regulation in zebrafish and changes in iron status have been shown to influence their expression levels [28]–[31]. Thus, we investigated the effect of different chelators on mRNA levels in adult fish in the gut, liver and heart tissue. Hepcidin is a multifunctional peptide and the chief iron regulatory hormone in vertebrates [29]–[30]. One of its functions is to bind to FPN, which is located on the basolateral surface of the enterocyte, and prevent iron absorption. Upon binding to FPN, hepcidin causes it to be broken down when the body’s iron supplies are adequate. DMT-1 functions as an iron transporter in vertebrates. It can also transport other divalent metals [28]. Adult fish were injected *i.p.* with 10 µL of 100 µM chelators and the gene expression at 24 h post-treatment was measured.

**Figure 6:**
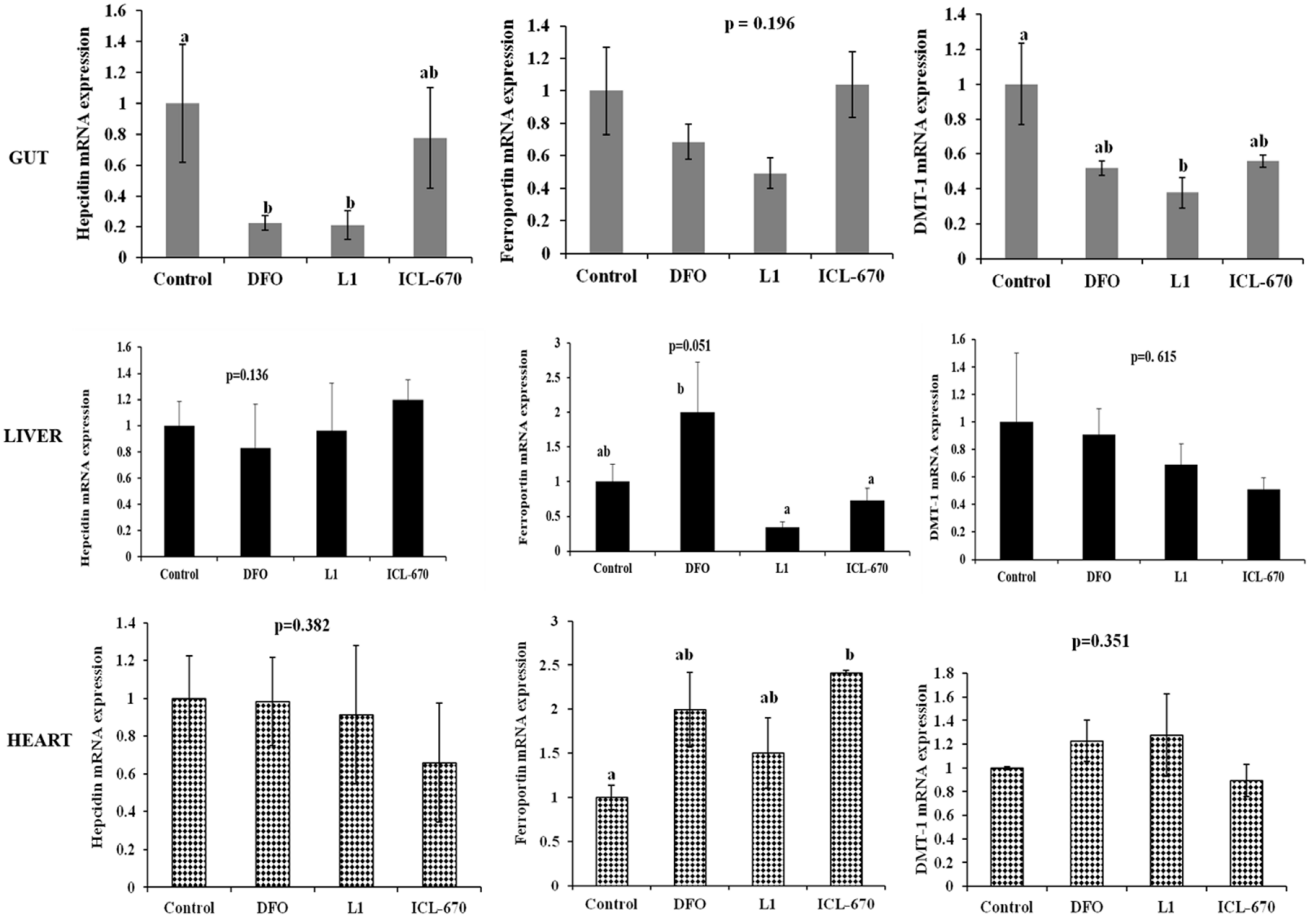
Gene expression of hepcidin, ferroportin (FPN) and divalent metal transporter (DMT-1) in the gut, liver and heart tissues of adult zebrafish after 24 h exposure to 100 µM (10 µL injection) of iron chelators. Hepcidin expression in the liver and heart tissue of adult zebrafish did not differ significantly from control. However, DFO and L1 significantly down-regulated hepcidin expression in the gut. There was no change in FPN expression in the gut upon chelator exposure. However, L1 and ICL-670 down-regulated, while DFO upregulated FPN in the liver and all chelators upregulated FPN in the heart. Treatment with DFO, L1 and ICL-670 resulted in a down-regulation of gut DMT-1. Gene expression values were normalized to β-actin and are presented as means ± SEM. Treatments that do not share a common letter are significantly different from each other, n = 6 for all treatments.

None of the iron chelators tested significantly altered the levels of hepcidin in the liver or heart. DFO and L1 caused a down-regulation of hepcidin expression in the gut. While ICL-670 also down-regulated hepcidin in the gut, its effect was less than that of DFO and L1 (**Figure 6**). Chelators did not significantly alter FPN expression in the gut. L1 and ICL-670 down-regulated, while DFO up-regulated FPN expression in the liver. All chelators caused a slight up-regulation of FPN in the heart with ICL-670 showing a significant difference from control. DFO, L1 and ICL-670 caused a down regulation of DMT-1 in the gut with L1 causing the strongest change. However, none of the iron chelators significantly altered DMT-1 expression in the liver and heart.

## Discussion

Iron chelators are used to reduce iron levels in patients that are susceptible to iron overload. Although a wide range of toxicities have been reported to occur, information on the effect of iron chelators in embryo development is lacking. Thus we investigated the effects of iron chelator exposure on mortality, hatching and morphology of zebrafish embryos. We also investigated possible mechanisms of toxicity by measuring the changes in cardiac function and the expression of hepcidin, FPN and DMT-1, genes anticipated to be sensitive to iron status.

Different chelators (DFO, L1, ICL-670) exerted distinct effects in zebrafish embryos and differed in the extent of toxicity exerted (**Figures 2** **to** **5**). While DFO treated embryos did not differ from control in any of the parameters measured; ICL-670 treated embryos exerted a range of toxicity which increased with increasing dose and longer duration of exposure. Exposure to L1 was associated with small changes to morphology.

The chemical properties of pharmacological agents and the characteristics of the zebrafish chorion may have separate, additive or synergistic effects on the chelator-induced toxicities. The size, polarity and specificity of each chelator for cations may vary and may influence the ability of chelators to permeate the zebrafish embryo, thus influencing the observed toxicities. The notable differences in size of chelators (DFO-560 Da, L1-139 Da and ICL-670-373 Da) and polarity may also be important factors influencing the intensity of chelator exposure in embryos [35]–[37]. Moreover, the hydrophilic nature of DFO may prevent it from readily entering cells to elicit this pattern of toxicity. A direct correlation has been reported for the rate of cellular uptake of a drug and its potential toxicity [37]. Direct injection might overcome this barrier compared to the soaked solutions as reported in a recent study on the evaluation of cardiotoxic drugs in zebrafish embryos [25], however the generality of this approach is not well-documented, and continuous exposure (up to 96 hpf in our case) may not be generated in the case of single direct injection. Further experimentation is needed to probe this.

Another important aspect of toxicity in relation to iron chelation therapy may be the removal or displacement of iron or essential other metals. In the absence of iron overload, iron chelators can interfere with zinc, copper and other micronutrient binding although the binding constants for these chelators to other metal ions are relatively small compared to Fe (III) (the log cumulative stability constant of DFO-Fe (III) is 30.6 versus 11.1 for DFO-Zn ^2+^) [38]. Additionally, reducing essential iron in the cell can result in reduced cell proliferation by inhibiting intracellular ribonucleotide reductase [39]. Although the precise daily iron requirements of teleost fish are unknown, the daily loss of iron is comparable to that in humans [40]. It has also been shown that developing fish embryos receive sufficient iron from maternal stores in the yolk and that iron acquisition by fish embryos is generally limited, while studies show that mature teleost fish can become iron limited [41]. Roeder and Roeder showed that retarded growth results when swordtail and platyfish were fed iron poor foods, with growth rates returning to normal when the diet was supplemented with iron salts [42]. Thus, although the effects of chelators were less pronounced in adults the significant change in heart function observed upon treatment with ICL-670 (**Figure 5**) may have resulted due to the depletion of essential cardiac iron or perturbation of metal balance (other essential metal ions) in heart tissue. Furthermore, the higher lipophilicity of ICL-670 has been shown to cause accumulation in some tissues and related organ damage in humans [43].

The seemingly mild effects of chelators on mRNA expression (**Figure 6**) are likely due to the short duration of exposure. Hepcidin is a multifunctional peptide with a key role in iron metabolism. FPN exports iron to the blood stream during absorption while DMT-1 functions as a carrier for most divalent metals across the apical surface of the cell. DFO produced a slight but insignificant increase in FPN expression after 24 h of exposure while gene expression in L1 and ICL-670 treated fish did not differ from control. Because DFO is known to be efficient at hepatocellular iron removal this finding is not surprising [1]. This may mean that DFO caused a reduction in the zebrafish liver iron causing an iron poor state.

DFO and L1 also induced the greatest change in hepcidin expression in the gut; causing a significant down-regulation of the gene compared to control, while ICL-670 did not appear to significantly affect hepcidin expression. When iron levels fall in the body, hepcidin levels are also decreased and more FPN is available to bring iron into the body and to release it from storage. Down regulation of hepcidin implies that cells are iron poor and causes enhanced iron absorption through increased FPN expression. This would be expected if these iron chelators chelate significant amounts of iron. Thus, it is likely that increasing the dose of chelator would result in a corresponding down regulation in hepcidin with the effect being increased iron absorption. Interestingly, ICL-670 did not have the same effect.

DMT-1 functions as an iron transporter in vertebrates. It can also transport other divalent metals. For example, rat DMT-1 has been shown to transport a range of divalent metal cations, including Fe, Pb, Zn, Cu and Cd [44]. DMT-1 has a critical function in iron metabolism as it allows the entrance of iron through the duodenal enterocyte, and enables the utilization of iron by cells via the transferring receptor mediated iron uptake. DMT-1 is also required for transporting iron out of the endosome and into the cytosol where it is incorporated into proteins or stored in ferritin [27].

The response of zebrafish to chemicals such as small molecules, drugs and environmental toxicants can be similar to that of mammals, however, it is important to conduct further studies that are aimed at determining how the effect of different iron chelator concentrations in zebrafish are related to those of mammalian models. This is especially relevant as the exposure to drugs in fish embryos is static, and internal concentrations are established by partition equilibrium. While in mammals, drugs are administered by single or repeat doses, and the exposure is not static. Thus, it is important to appropriately translate all findings obtained in experimental models. Koren and Ito [45] provide an excellent review regarding this.

Not surprisingly, juvenile zebrafish were more susceptible to toxic effects than adults. This may have resulted from the longer duration of chelator exposure (96 hpf) in the embryos compared to the single dose given to adult zebrafish. The short duration of exposure in adults may also account for the mild alterations in gene expression observed in adults in the organs tested. This study provides information on the feasibility, potential doses of interest and time effects for testing chelator toxicity in zebrafish. Further studies of gene expression which take advantage of increased exposure time in adults (chronic exposure) would help to improve our understanding.

## Conclusions

This study demonstrates that clinically approved iron chelators vary in their ability to induce toxicity in zebrafish embryos and adults, and that the time and dose of exposure are major factors influencing toxicity. DFO exposure did not induce any toxicity; there was no change in mortality, morphology, or hatching rate. L1 did not significantly affect mortality but caused morphological alterations at higher concentrations. While ICL-670 caused significant morphological deformities and hatching problems in zebrafish embryos above certain concentrations such that these iron chelators are influencing the development into larvae. Unlike other chelators, ICL-670 caused significant change in the heart rate and cardiac output in adult zebrafish when injected at a concentration of 100 µM (10 µL). Changes in expression of hepcidin, FPN and DMT-1 genes were observed in adults, however, the effect are not pronounced as in embryos.

It is most likely that a combination of factors including the differential permeability of the components of the embryo; the size, lipophilicity and chemical structure of DFO, L1 and ICL-670 influenced their ability to induce toxic effects in zebrafish. Studies focused on characterizing the permeability barriers in the zebrafish chorion will further elucidate the factors that influence the observed chelator toxicity. Additionally, future studies which investigate the method of chelator exposure, as well as the absorption, metabolism and excretion profile of each chelator in zebrafish are recommended. Such studies can elucidate scaling factors which may allow more suitable and accurate comparisons and correlation to rodents, and other relevant experimental models.

## Supporting Information

File S1 **Additional data is given in supplementary files Figure S1 to Figure S7.**
(DOCX)
