# Supplementary material for: Clinically Approved Iron Chelators Influence Zebrafish Mortality, Hatching Morphology and Cardiac Function
Source: PLoS One. 2014 Oct 16;9(10):e109880. doi: 10.1371/journal.pone.0109880 (PMC4199627; doi:10.1371/journal.pone.0109880)
Supplement: File S1 — Additional data is given in supplementary files Figure S1 to Figure S7. (DOCX) [file pone.0109880.s001.docx]

**Clinically Approved Iron Chelators Influence Zebrafish Mortality, Hatching Morphology and Cardiac Function**

Jasmine L. Hamilton^1^, Azadeh Hatef ^2^, Muhammad Imran ul-haq^1^, Neelima Nair ^2^, Suraj Unniappan ^2^ and Jayachandran N. Kizhakkedathu^1,3^

**Supporting Information**

**Figure S1:** ^1^H NMR spectrum of synthesized ICL-670. Spectrum is similar to that reported in literature (32).

**Figure S2:** ESI-MS spectrum of synthesized ICL-670. Mass value matches with the theoretical calculation.

**
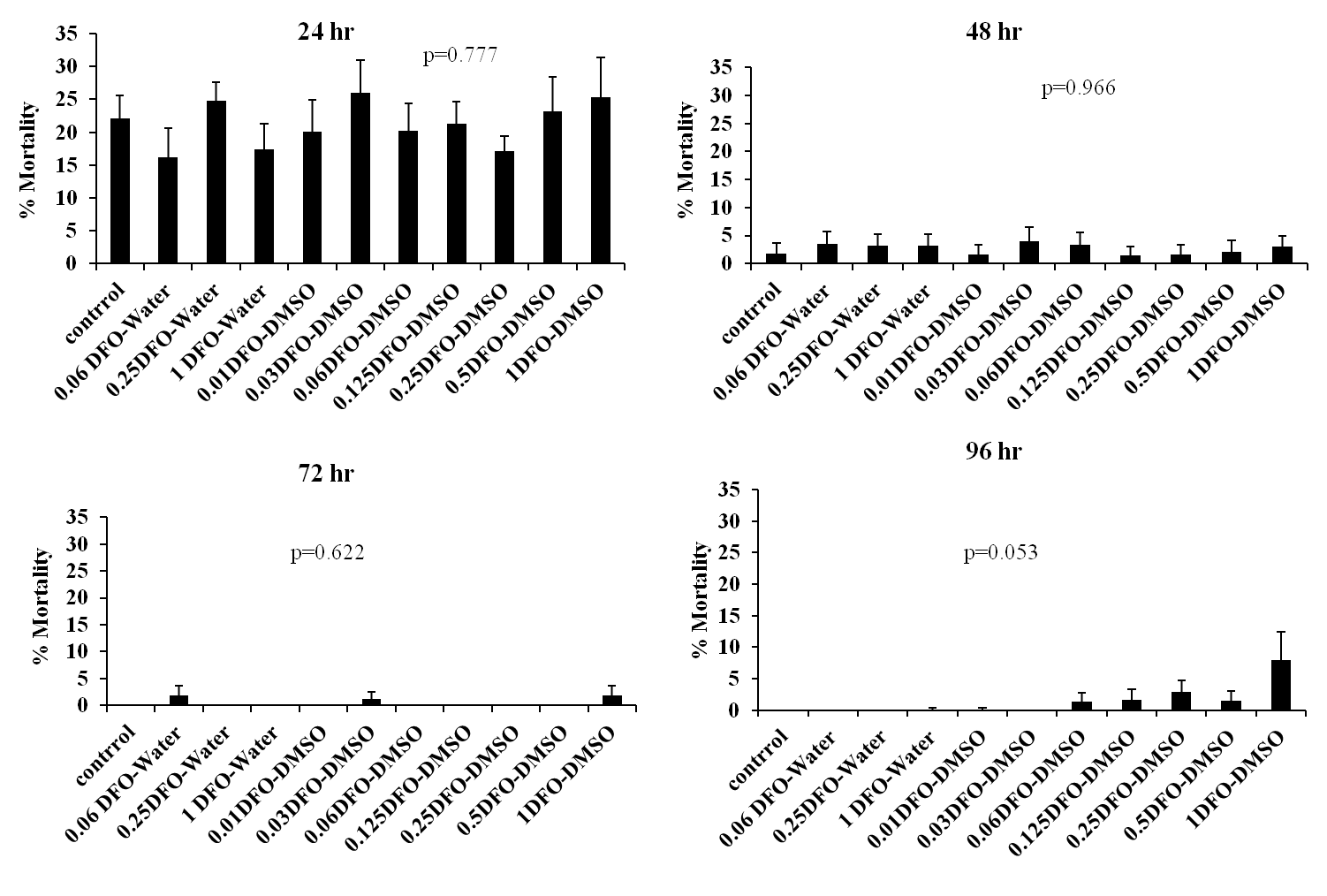
**

**
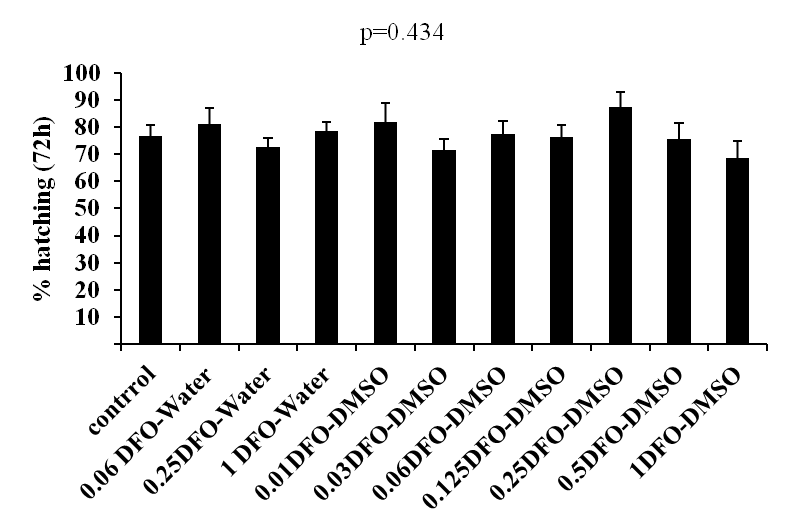
**

**Figure S3: DMSO does not interfere with toxicity of embryos.** To determine whether DMSO influenced or enhanced the toxicity of chelators, embryos were exposed to DFO (0-1mM) dissolved in either fish water (control) or DMSO for 96 hpf. Embryos did not differ significantly in mortality rates or hatching success throughout the duration of the study.

**
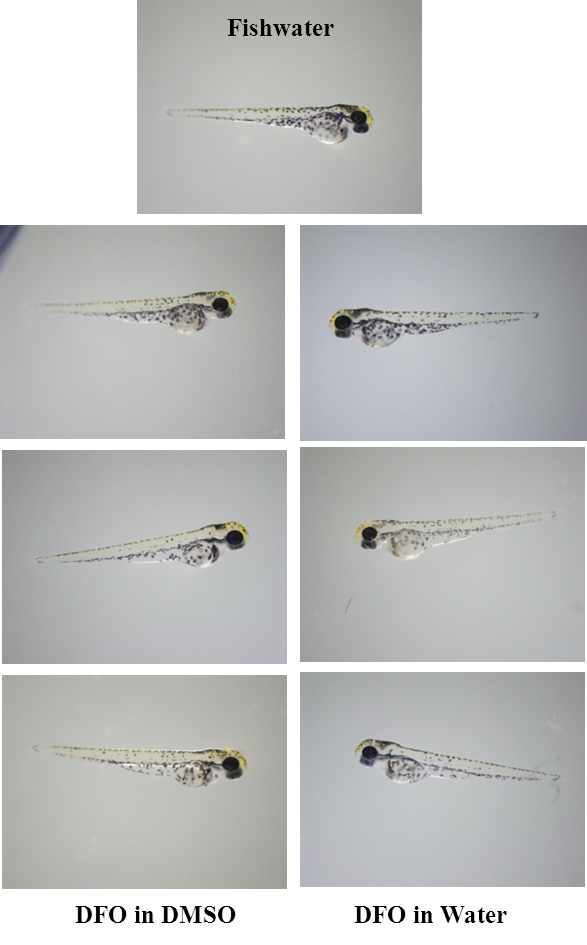
**

**Figure S4: DMSO does not influence the morphology of zebrafish embryos.** To determine whether DMSO influenced or enhanced the toxicity of chelators, embryos were exposed to DFO (0-1mM) dissolved in either fish water (control) or DMSO and their morphology was observed for 96 hpf. The morphology of zebrafish embryos exposed to DFO in fishwater did not differ from the morphology of zebrafish embryos exposed to DFO dissolved in DMSO.


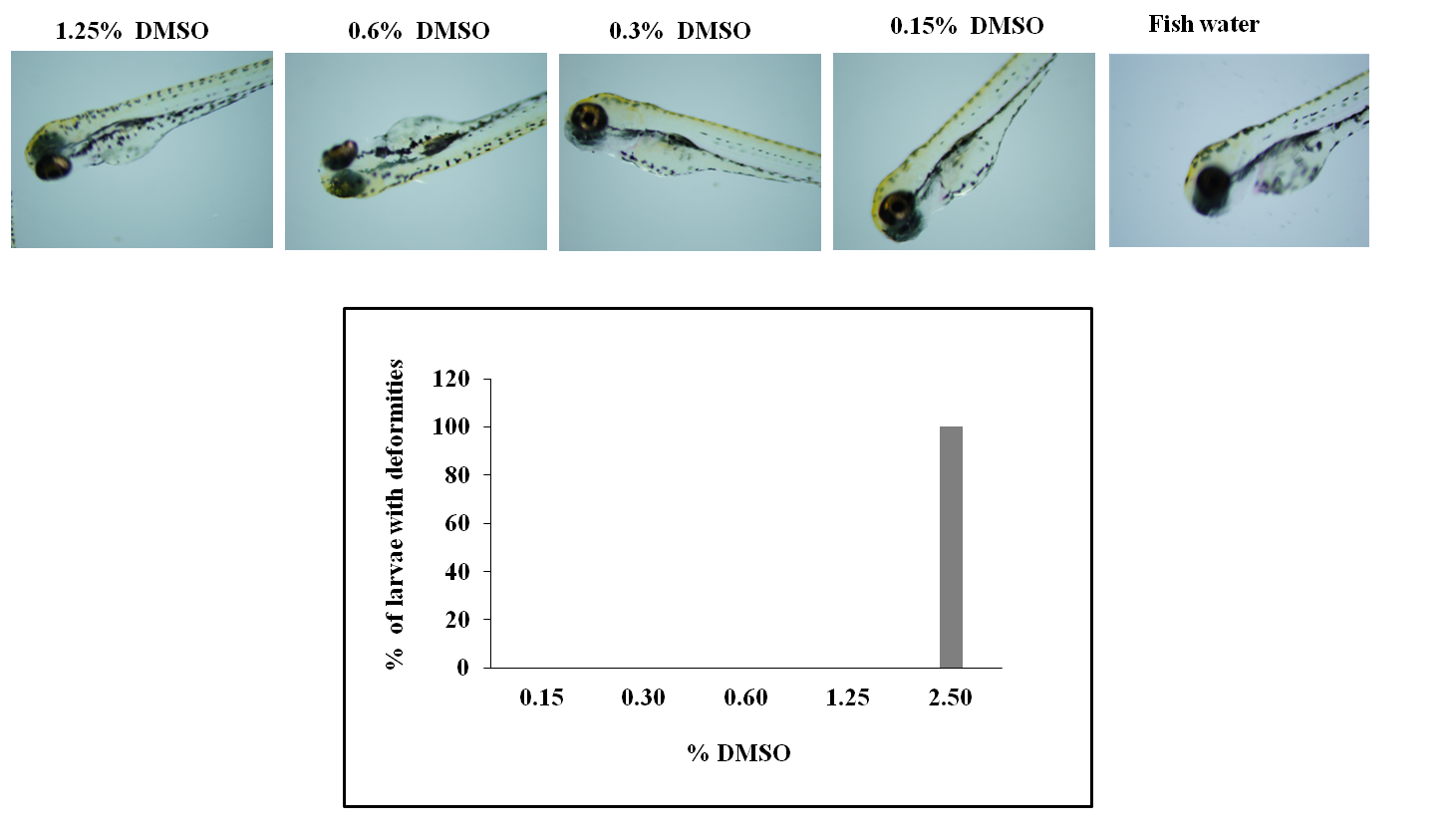


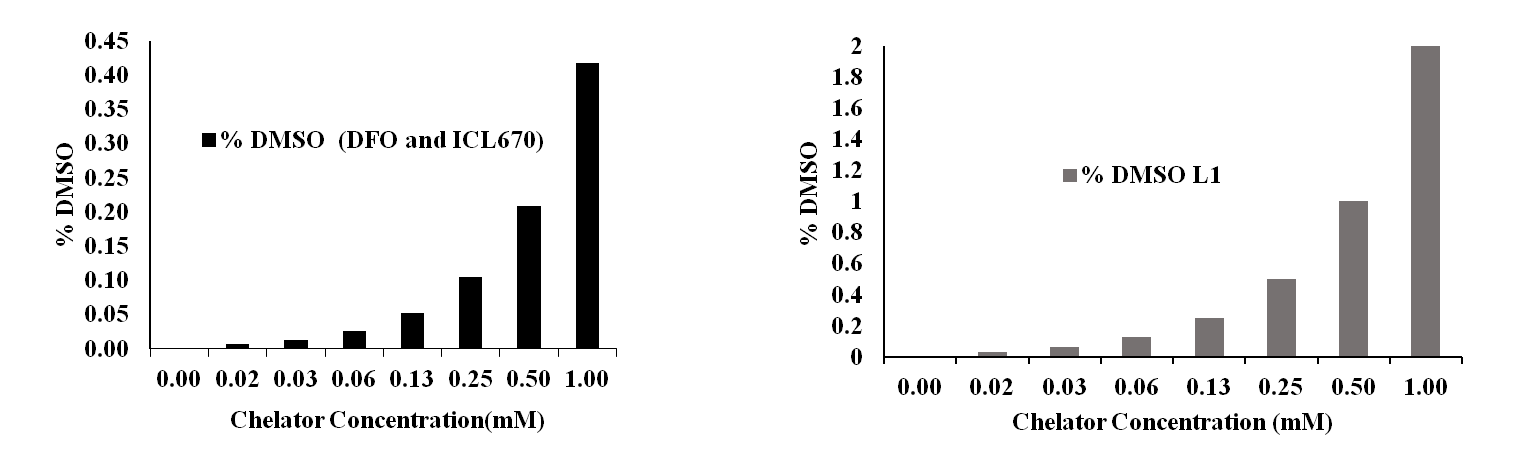


**Figure S5:** To ensure that the amount of carrier solvent used did not influence the observed effects, DMSO was titrated and the effects on morphology were observed. The morphology of DMSO treated embryos after 96 hpf exposure and the concentration of DMSO present per concentration of each chelator.


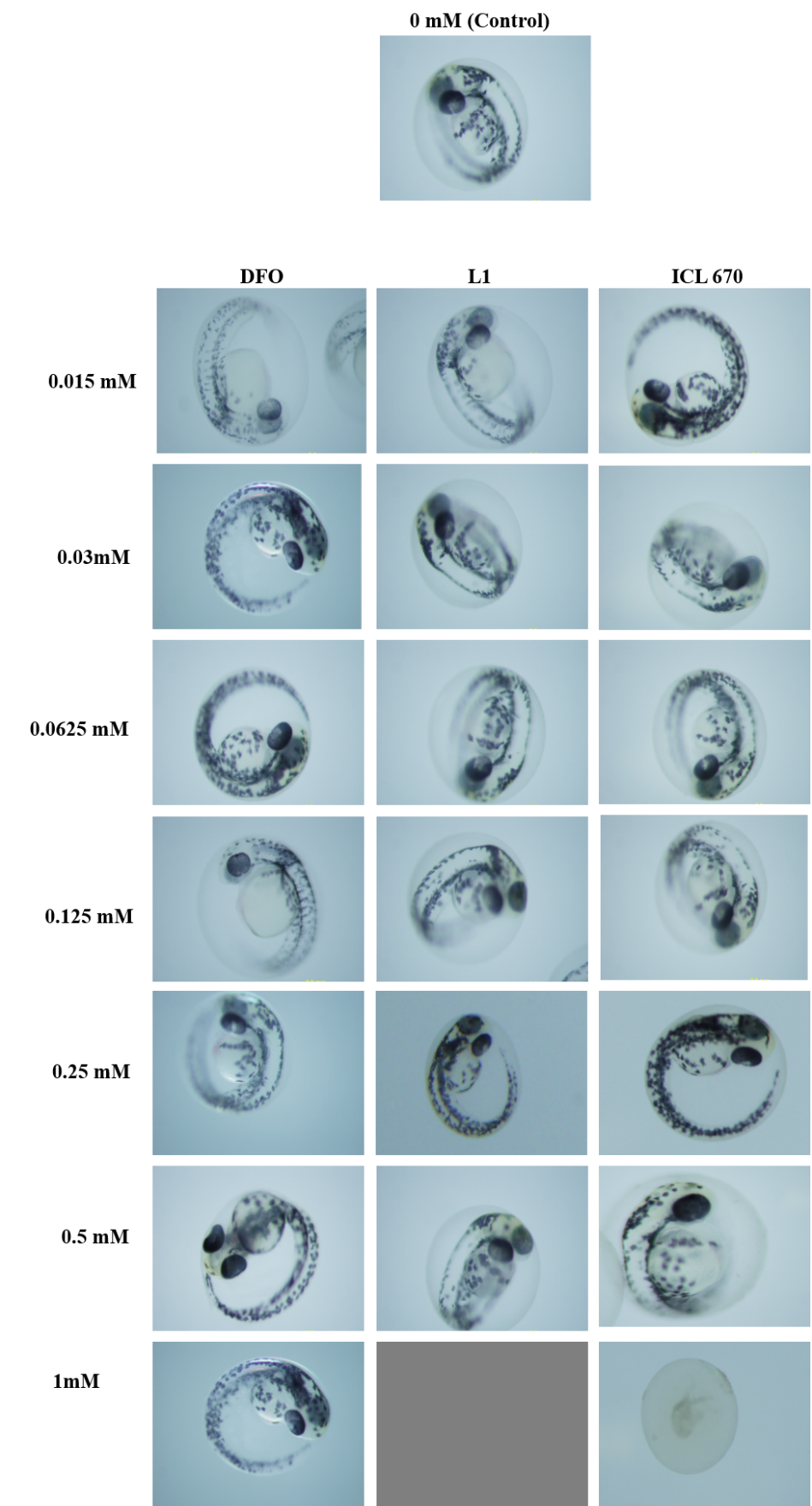


**Figure S6:** Morphology of Zebrafish embryos after 48 hpf of exposure to DFO, L1 and ICL-670. Zebrafish kept in DMSO were used as controls. No abnormalities were observed in DFO treated embryos up to concentrations of 1mM. However; 48 hpf exposure to L1 and ICL670 were associated with developmental abnormalities and mortality at concentrations above 0.5 mM.


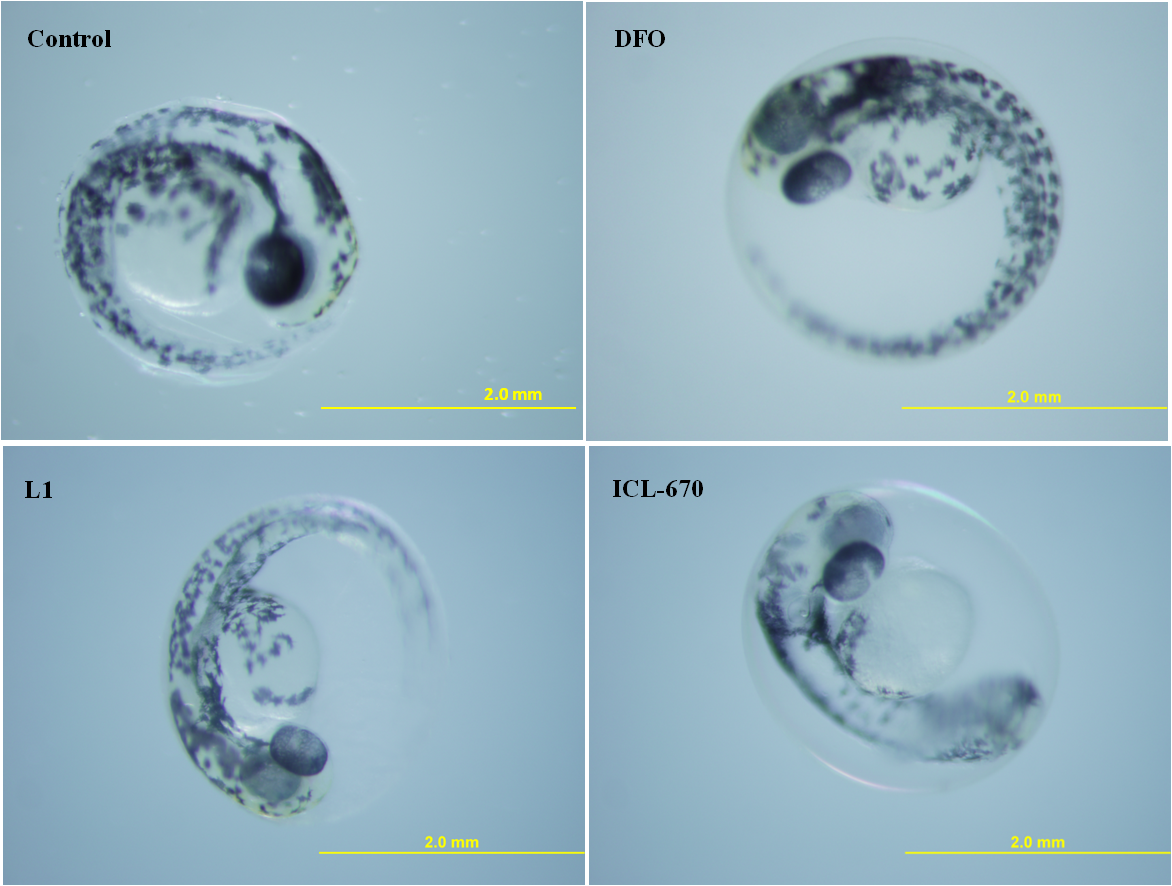


**Figure S7: Malformations of zebrafish embryos exposed to 0.5 mM iron chelators.** Representative optical images of zebrafish embryo morphology after 48 hpf of exposure to DFO, L1 and ICL 670. Zebrafish kept in DMSO was used as control. No abnormalities were observed in DFO nor L1 treated embryos up to concentrations at 0.5mM after 48 hpf treatment.
